# Which Emergency Medicine Milestone Sub-competencies are Identified Through Narrative Assessments?

**DOI:** 10.5811/westjem.2019.12.44468

**Published:** 2019-12-20

**Authors:** David Diller, Shannon Cooper, Aarti Jain, Chun Nok Lam, Jeff Riddell

**Affiliations:** * LAC+USC Medical Center, Keck School of Medicine of the University of Southern California, Department of Emergency Medicine, Los Angeles, California; † Henry Ford Allegiance Health, Department of Emergency Medicine, Jackson, Michigan

## Abstract

**Introduction:**

Evaluators use assessment data to make judgments on resident performance within the Accreditation Council for Graduate Medical Education (ACGME) milestones framework. While workplace-based narrative assessments (WBNA) offer advantages to rating scales, validity evidence for their use in assessing the milestone sub-competencies is lacking. This study aimed to determine the frequency of sub-competencies assessed through WBNAs in an emergency medicine (EM) residency program.

**Methods:**

We performed a retrospective analysis of WBNAs of postgraduate year (PGY) 2–4 residents. A shared mental model was established by reading and discussing the milestones framework, and we created a guide for coding WBNAs to the milestone sub-competencies in an iterative process. Once inter-rater reliability was satisfactory, raters coded each WBNA to the 23 EM milestone sub-competencies.

**Results:**

We analyzed 2517 WBNAs. An average of 2.04 sub-competencies were assessed per WBNA. The sub-competencies most frequently identified were multitasking, medical knowledge, practice-based performance improvement, patient-centered communication, and team management. The sub-competencies least frequently identified were pharmacotherapy, airway management, anesthesia and acute pain management, goal-directed focused ultrasound, wound management, and vascular access. Overall, the frequency with which WBNAs assessed individual sub-competencies was low, with 14 of the 23 sub-competencies being assessed in less than 5% of WBNAs.

**Conclusion:**

WBNAs identify few milestone sub-competencies. Faculty assessed similar sub-competencies related to interpersonal and communication skills, practice-based learning and improvement, and medical knowledge, while neglecting sub-competencies related to patient care and procedural skills. These findings can help shape faculty development programs designed to improve assessments of specific workplace behaviors and provide more robust data for the summative assessment of residents.

## INTRODUCTION

In 2012, the Accreditation Council for Graduate Medical Education (ACGME) developed educational milestones to serve as the primary framework for competency-based assessment in graduate medical education.^1^ These educational milestones were framed within specialty-specific sub-competencies, with each sub-competency belonging to one of six previously established ACGME core competencies.^2^ A central tenet to the milestones framework is the emphasis on resident trainee assessment based on observable performance and behaviors.^3^ While many workplace-based assessment strategies have been piloted, a comprehensive validated approach to resident assessment within the milestones framework has yet to be developed.^4^–^6^

Workplace-based narrative assessment (WBNA), also known as the in-training evaluation report (ITER),^7^ uses descriptive commentary for performance assessment and has been proposed as an alternative method to checklists and rating scales.^7^–^9^ Through descriptive commentary, WBNA provides assessors with a version of assessment without the constraints of pre-selected ratings or options, theoretically allowing for a more robust analysis. WBNA can exist independently as an evaluation form, or in combination with checklists or rating scales as a hybrid evaluation model.

The benefits of WBNAs in medical education are well documented. WBNAs have been shown to be useful in ranking trainees,^10^ detecting learners who are experiencing difficulty,^11^ identifying milestone sub-competencies that are more difficult to assess,^10^–^12^ and predicting the need for resident remediation.^13^ In addition to providing a richer data source, narrative assessments are also appreciated by learners.^14^,^15^ Whether used in addition to anchor-based rating tools^16^ or as an independent assessment method, descriptive commentary can be a reliable method of assessment that influences faculty judgment on global resident performance.^7^

Despite these benefits, validity evidence for using WBNAs as a method for assessing milestone sub-competencies in graduate medical education is lacking. The prevalence of vague comments, such as “hard worker” and “pleasant to work with,” are well-documented,^17^–^21^ and it is unclear how beneficial these comments are in assessing learners within the milestones framework. Furthermore, while contextual framing and faculty development can provide more robust narrative assessments in terms of both quantity and quality of comments,^9^ the frequency with which WBNAs comment on specific milestone sub-competencies remains unknown. Without knowing which milestone sub-competencies are being assessed, program directors and clinical competency committees (CCC) may be left to assume competence in a broad range of skills, despite a lack of explicit evidence to support those conclusions.^8^

This study aims to determine the frequency of milestone sub-competencies assessed through semi-annual WBNAs in an emergency medicine (EM) residency program.

## METHODS

### Settings and Participants

Faculty at the LAC+USC EM residency program complete semi-annual WBNAs on residents with whom they have worked over the prior six-month period. An internally created online form through the education management platform MyEvaluations allows faculty to provide descriptive responses to two prompts: “Please describe at least one area of strength for this resident” and “Please describe at least one area for potential improvement for this resident.” Faculty WBNAs are encouraged but not mandatory, and not all faculty complete WBNAs on all residents. No formal training exists for faculty regarding milestone sub-competencies or workplace-based assessment strategies.

**Educational Research Capsule Summary**

What do we already know about this issue?

*Narrative assessments are a commonly used evaluation tool for making judgments on resident clinical performance*.

What was the research question?

What milestone sub-competencies are assessed through narrative assessments in an EM residency program?

What was the major finding of the study?

*Unstructured narrative assessments identified relatively few milestone sub-competencies*.

How does this improve population health?

*These results can improve evaluation tool design and faculty development to improve the validity for narrative assessments within the Milestones framework*.

### Study Design

We performed a retrospective analysis of the WBNAs of postgraduate year (PGY) 2–4 residents completed between the second semester of 2016 and the first semester of 2017. WBNAs of PGY-1 residents and second semester PGY-4 residents were excluded due to limited faculty contact with PGY-1 residents and a hypothesized concern from the study authors regarding a lack of critical assessment of PGY-4 residents during their final semester of training. The local institutional review board determined the study was exempt.

### Protocol

Author DD collated, de-identified, and randomized the selected WBNAs. Three authors (SC, AJ, JR), blinded to both the identity of the faculty assessor and resident being assessed, reviewed the WBNAs to determine whether the comments assessed any of the 23 EM-specific milestone sub-competencies.^22^ Prior to reviewing narrative assessments, the study authors SC, AJ, and JR met to establish a shared mental model by reading and discussing the ACGME milestones framework. We reviewed the first 50 WBNAs, resolved discrepancies as a group, and developed a guide detailing our interpretations of the milestones (Appendix A). We reviewed subsequent blocks of 50 WBNAs, discussed discrepancies, and updated our guide in an iterative manner. This process continued until the inter-rater reliability between the three reviewers was good (k > 0.8). We then independently reviewed the remaining WBNAs and recorded the sub-competencies that each WBNA assessed in spreadsheets that included all 23 EM-specific milestone sub-competencies. For any given WBNA, there was no limit to the number of sub-competencies that could be assessed (Table 1).

### Analysis

We performed descriptive statistics and conducted chi-square and analysis of variance tests for comparison on milestone proportions and means across three faculty levels to determine whether milestone sub-competencies were reported more frequently by specific faculty cohorts based on years of experience. All two-tailed significance tests were computed in Stata 13 with a set to 0.05 (StataCorp. 2013. Stata Statistical Software: Release 13. College Station, TX: StataCorp LP).

## RESULTS

During the 2016–2017 study period, we analyzed 2517 WBNAs. WBNAs were completed for 51 PGY 2–4 residents by 61 faculty members. Each resident received an average of 49 WBNAs (range: 37 to 71), and each faculty member completed an average of 41 WBNAs (range: 1 to 102). From the 2517 WBNAs, we identified a total of 5130 milestone sub-competencies, with an average of 2.04 milestone sub-competencies assessed per WBNA. Of the 23 EM milestone sub-competencies, those most frequently identified through WBNAs were multitasking, medical knowledge, practice-based performance improvement, patient-centered communication, and team management. The sub-competencies least frequently identified through WBNAs were pharmacotherapy, airway management, anesthesia and acute pain management, goal-directed focused ultrasound, wound management, and vascular access. Overall, the frequency with which WBNAs assessed individual sub-competencies was low, with 14 of the 23 sub-competencies being assessed in less than 5% of WBNAs (Range: 0–33.3%) (Figure).

Junior faculty, defined as attending physicians in practice for less than five years, represented only 16% of the faculty, but completed 24% of the WBNAs. Conversely, senior faculty, defined as attending physicians in practice for greater than 15 years, represented 26% of the faculty, but completed only 17% of the WBNAs. Mid-career faculty, defined as attending physicians in practice between 5–15 years, accounted for the 57% of the faculty and 58% of the WBNAs. On average, junior faculty members identified 2.30 milestone sub-competencies per WBNA, compared to 2.03 milestone sub-competencies per WBNA for mid-career faculty, and 1.88 milestone sub-competencies per WBNA for senior faculty.

There was a statistically significant difference in milestone sub-competencies identified by faculty cohorts based on years of experience (ie, junior, mid-career, senior) in 14 of the 23 EM milestone sub-competencies (Table 2). On average, senior faculty tended to identify fewer individual milestone sub-competencies on WBNAs when compared to their junior or mid-career faculty colleagues.

## DISCUSSION

In this evaluation of 2517 WBNAs at a single residency program, we found that each WBNA on average identified two milestone sub-competencies, with WBNAs clustering around five specific sub-competencies and largely ignoring 60% of the sub-competencies. All sub-competencies are directly observable in the clinical environment,^22^ and while certain sub-competencies such as medical knowledge can be observed elsewhere (eg, in-service examination, mock oral boards), those related to patient care and procedural skills that are best observed in the clinical environment were notably absent from the WBNAs. While junior faculty narratives assessed slightly more sub-competencies than mid-career or senior faculty narratives, the overall frequency of addressing milestone sub-competencies through WBNAs was low.

These findings are concerning because when WBNAs fail to comment on the majority of milestone sub-competencies, program directors and CCCs are left to make judgments regarding resident performance on a wide range of unassessed skills.^8^ This is detrimental to resident education, as the assumption of competence limits future targeted observations and interventions by faculty members, and it may either suppress a resident’s desire to self-report areas of weakness or it may promote a sense of inappropriate overconfidence when true performance lags behind resident self-assessment.

Despite the value of WBNAs as an assessment tool,^7^–^9^ we hypothesize a lack of consistent faculty development as one reason for faculty’s poor performance in identifying numerous milestone sub-competencies. According to van der Vleuten et al.,^23^ as an instrument seeks to assess higher levels on Miller’s pyramid,^24^ the validity is more dependent on the assessors and the quality of the implementation as opposed to the instrument itself. Workplace-based observation and assessment of resident performance, and the subsequent narrative documentation of these observations and interpretations, is a skill that requires both training and practice. Too often, assessors receive little to no training in the practice of delivering WBNAs,^6^ even though studies suggest that faculty development can improve the number and quality of narrative comments.^9^ While no recurrent faculty training program exists within our department, we do not know whether prior faculty development initiatives had been implemented in past years. Regardless, the fact that junior faculty narratives assessed slightly more sub-competencies than mid-career or senior faculty narratives suggests that if there were prior faculty development initiatives, they did not have a lasting effect.

Faculty were more likely to comment on sub-competencies relating to the ACGME core competencies of interpersonal and communication skills, practice-based learning and improvement, and medical knowledge, than on sub-competencies relating to patient care and procedural skills. This differs from prior studies conducted in general surgery and internal medicine training programs, which demonstrated a higher frequency of faculty comments regarding patient care and greater variability in comments regarding interpersonal communication skills.^25^,^26^ The difference in ACGME core competencies identified through WBNAs between our study and prior studies may be due to differing specialty-specific, faculty-resident dynamics, varying prompts and constructs of the WBNAs used, faculty training in workplace-based assessment, or cultures inherent to respective specialties or institutions.

We found that junior faculty completed WBNAs more frequently than senior faculty and their WBNAs identified milestone sub-competencies more frequently. It is unclear why this may be; however, one explanation may be that junior faculty members are more familiar with the milestones framework than senior faculty. Additionally, junior faculty generally work more shifts than senior faculty, and thus may be more likely to observe and comment on observed behaviors.

## LIMITATIONS

Our study had several notable limitations. It is a single-center, specialty-specific study, which limits its generalizability. The fact that we did not account for the number of shifts worked per faculty member limited our ability to assess for whether shift count influenced the differences among faculty cohorts. We did not account for faculty members who provided the same verbatim written commentary for each WBNA, regardless of resident performance, which was an observed practice. While this type of behavior may alter the overall frequencies of milestone sub-competencies our study identified, as well as the quality of the assessment provided, we chose to include their data because both residents and the CCC receive their comments on an individual level.

The WBNAs used at our institution did not include any prompting for faculty to comment on specific milestone sub-competencies, which may have resulted in lower frequencies of milestone sub-competencies identified. As a result, these findings may not be generalizable to institutions that use evaluation structures with specific milestone prompts. In addition, the lack of an annual formalized faculty training raises the question as to whether consistent faculty development would improve the frequency of milestone sub-competencies identified.

Finally, while we interpreted the WBNAs based on definitions and consensus, evaluators often “read between the lines” of narratives when providing summative assessments on residents.^8^,^27^ Therefore, identifying the frequency of milestone sub-competencies may undervalue the role of WBNAs in providing information for summative assessments. However, we would contend that a more analytical process than evaluator gestalt is necessary for improved reliability and validity in providing competency judgments on trainees.

We recognize that identifying milestone sub-competencies is not the only measure in determining the quality of an assessment. Similar to prior research,^26^ many WBNAs commented on non-ACGME themes. While this study was not designed to evaluate these comments, prior research has demonstrated their value to both faculty^27^ and residents.^7^ However, if assessment of individual sub-competencies is desirable,^8^,^28^ targeted faculty development activities can be implemented to enhance sub-competency identification. With improved assessments that target previously unaddressed milestone sub-competencies, CCCs and program directors will have better guidance towards providing summative assessments regarding resident performance.^8^

Future research should examine the effectiveness of these faculty development programs in improving the frequency of milestone sub-competencies identified, as well as evaluating for satisfaction of both residents and faculty members post-intervention. In addition, given the disparities in assessments of male and female residents,^29^ it is important to examine the role that gender (of both the assessor and trainee) plays in identifying which sub-competencies are identified through WBNAs. Finally, while we believe our coding guide is generalizable to other EM programs interested in mapping WBNAs to the milestones, it is possible that natural language processing, which aims to program machines to interpret human language,^30^ could replace the need for manual assessment of WBNAs. Future research could look at the feasibility of natural language processing in the evaluation of WBNAs.

## CONCLUSION

Our study demonstrates that unstructured WBNAs identify relatively few milestone sub-competencies. Faculty tend to assess similar sub-competencies related to interpersonal and communication skills, practice-based learning and improvement, and medical knowledge, while neglecting sub-competencies related to patient care and procedural skills. These findings can help shape faculty development programs designed to improve assessments of specific workplace behaviors and provide more robust data in the summative assessment of residents.

## Supplementary Information



## Figures and Tables

**Figure:**
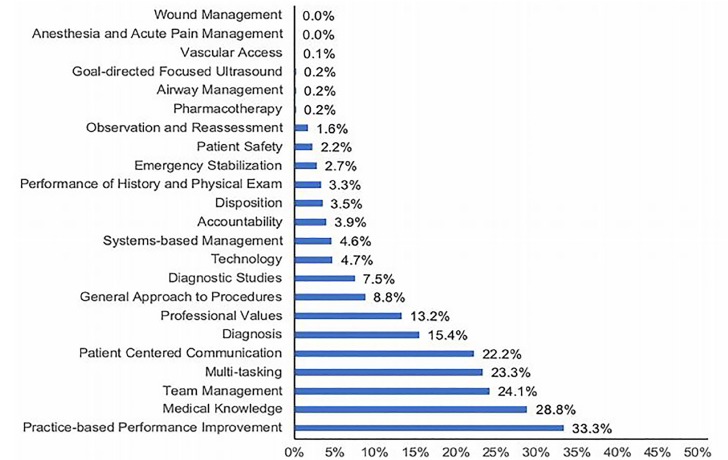
Frequency of emergency medicine milestone sub-competencies identified per workplace-based narrative assessment.

**Table 1:** Example of a workplace-based narrative assessment identifying emergency medicine milestone sub-competencies.

Workplace-Based Narrative Assessment	Milestone(s) Assessed
“She has a great fund of knowledge, advocates for her patients, and does a great job managing the critically ill.”	Medical Knowledge Patient Centered Communication Emergency Stabilization
I love working with him. He’s very friendly, humble, hard-working, and enjoys learning.”	None applicable

**Table 2:** Frequency of emergency medicine (EM) milestone sub-competencies identified by respective faculty assessor cohorts.

Emergency Medicine Milestone Sub-Competencies	Junior (<5 years)	Mid-Career (5–15 years)	Senior (>15 years)	p-value
Observation and reassessment	0.91%	2.54%	0.00%	<0.001
Disposition	2.28%	5.30%	0.16%	<0.001
Patient safety	4.33%	2.27%	0.64%	<0.001
Systems-based management	8.88%	4.81%	0.96%	<0.001
Diagnostic studies	12.07%	8.73%	1.44%	<0.001
Diagnosis	17.08%	17.47%	9.29%	<0.001
General approach to procedures	2.73%	5.23%	21.31%	<0.001
Patient-centered communication	14.12%	24.35%	23.08%	<0.001
Practice-based performance improvement	46.01%	27.03%	38.78%	<0.001
Multitasking	20.05%	25.86%	19.71%	0.002
Team management	30.07%	23.66%	20.99%	0.002
Medical knowledge	35.76%	27.10%	27.72%	0.002
Professional values	18.22%	12.24%	12.02%	0.003
Accountability	5.92%	3.78%	2.72%	0.028
Performance of history and physical exam	2.05%	4.06%	2.56%	0.056
Vascular access	2.00%	1.00%	0.00%	0.073
Airway management	0.68%	0.14%	0.16%	0.109
Emergency stabilization	3.87%	2.68%	1.92%	0.155
Goal-directed focused ultrasound	0.46%	0.14%	0.00%	0.176
Pharmacotherapy	0.00%	0.28%	0.00%	0.231
Technology	3.87%	5.23%	4.01%	0.325
Anesthesia and acute pain management	0.00%	0.00%	0.00%	-
Wound management	0.00%	0.00%	0.00%	-
