# Supplementary material for: Which Emergency Medicine Milestone Sub-competencies are Identified Through Narrative Assessments?
Source: West J Emerg Med. 2019 Dec 20;21(1):173–9. doi: 10.5811/westjem.2019.12.44468 (PMC6948702; doi:10.5811/westjem.2019.12.44468)
Supplement: Supplementary file 1 [file wjem-21-173-s001.pdf]

EM Milestone	Informal Name	Formal Explanation	Our Interpretation
	Not Applicable to Milestones sub-competencies	Vague comments that do not fit into a particular category	Good clinician, clinically strong, clinically solid, good personality, fun, good energy, enjoyable to work with, friendly, calm, nice, good teacher, humility, reach out your juniors, easy to get along with, hard working, kind, manages patients well, clinical skills, "think about how you would practice in the community", people look up to you, good judgment, good decision-making, good instincts, enthusiasm for learning, enjoys learning, pushing yourself, improving yourself
1	Stabilization	Emergency Stabilization (PC1) Prioritizes critical initial stabilization action and mobilizes hospital support services in the resuscitation of a critically ill or injured patient and reassesses after stabilizing intervention.	recognizes vitals, manages unstable/critical patient, recognizes need to intervene, determines sick v. not sick for their own patients
2	H&P	Performance of Focused History and Physical Exam (PC2) Abstracts current findings in a patient with multiple chronic medical problems and, when appropriate, compares with a prior medical record and identifies significant differences between the current presentation and past presentations.	thorough H&P, reliable exam, NOT thorough
3	Diagnostic Studies	Diagnostic Studies (PC3) Applies the results of diagnostic testing based on the probability of disease and the likelihood of test results altering management.	thorough workup, over-testing, good plans (double code plan)
4	Diagnosis	Diagnosis (PC4) Based on all of the available data, narrows and prioritizes the list of weighted differential diagnoses to determine appropriate management.	differential diagnosis, not anchoring, premature closure, good plans, NOT dipso, "clinical decisions, clinical instincts, clinical judgment, clinical gestalt, clinical acumen, Comfortable assessing patients and prioritizing them appropriately,
5	Pharm	Pharmacotherapy (PC5) Selects and prescribes, appropriate pharmaceutical agents based upon relevant considerations such as mechanism of action, intended effect, financial considerations, possible adverse effects, patient preferences, allergies, potential drug-food and drug-drug interactions, institutional policies, and clinical guidelines; and effectively combines agents and monitors and intervenes in the advent of adverse effects in the ED.	
6	Reassessment	Observation and Reassessment (PC6) Re-evaluates patients undergoing ED observation (and monitoring) and using appropriate data and resources, determines the differential diagnosis and, treatment plan, and displosition.	check back in on sick patients, good follow through with patients

7	Dispo	Disposition (PC7) Establishes and implements a comprehensive disposition plan that uses appropriate consultation resources; patient education regarding diagnosis; treatment plan; medications; and time and location specific disposition instructions.	Appropriate admission/discharge. Discharge instructions.
8	Multi-tasking	Multi-tasking (Task-switching) (PC8) Employs task switching in an efficient and timely manner in order to manage the ED.	efficiency, ability to move, fast, flow (apart from departmental level)- if not specified to department, flow defaults to multi-tasking, "i.e. manages north well" - more toward individual and not utilizing other resources, take COMMAND of the area, "controls the area well"
9	Procedures	General Approach to Procedures (PC9) Performs the indicated procedure on all appropriate patients (including those who are uncooperative, at the extremes of age, hemodynamically unstable and those who have multiple co-morbidities, poorly defined anatomy, high risk for pain or procedural complications, sedation requirement), takes steps to avoid potential complications, and recognizes the outcome and/or complications resulting from the procedure.	general procedural chit chat procedure not otherwise mentioned, EXCLUDING Airway, Pain management, vascular access, wound management, ultrasound
10	Airway	Airway Management (PC10) Performs airway management on all appropriate patients (including those who are uncooperative, at the extremes of age, hemodynamically unstable and those who have multiple co-morbidities, poorly defined anatomy, high risk for pain or procedural complications, sedation requirement), takes steps to avoid potential complications, and recognize the outcome and/or complications resulting from the procedure.	Airway, intubation
11	Anesthesia & Acute Pain Management	Anesthesia and Acute Pain Management (PC11) Provides safe acute pain management, anesthesia, and procedural sedation to patients of all ages regardless of the clinical situation.	Pain meds, sedation
12	Ultrasound	Other Diagnostic and Therapeutic Procedures: Goal-directed Focused Ultrasound (Diagnostic/Procedural) (PC12)	US- NOT US guided lines
13	Wound Management	Other Diagnostic and Therapeutic Procedures: Wound Management (PC13) Assesses and appropriately manages wounds in patients of all ages regardless of the clinical situation.	Lac repair, burns, irrigation, dressings, cosmesis

14	Vascular Access	Other Diagnostic and Therapeutic Procedures: Vascular Access (PC14) Successfully obtains vascular access in patients of all ages regardless of the clinical situation.	Central lines
15	Knowledge	Medical Knowledge (MK) Demonstrates appropriate medical knowledge in the care of emergency medicine patients.	Smart, knowledgeable, good fund of knowledge, in-service/boards, DOES NOT INCLUDE build your knowledge base or keep reading, bright
16	Safety	Patient Safety (SBP1) Participates in performance improvement to optimize patient safety.	sign out, shortcuts, QI, dangerous workups, don't go too quickly
17	Systems	Systems-based Management (SBP2) Participates in strategies to improve healthcare delivery and flow. Demonstrates an awareness of and responsiveness to the larger context and system of health care.	Overordering tests, managing flow of dept (specfically- NOT pod), fasttrack patients, utilizing other resources/staff/systems to improve flow, cost-effective care, but NOT "think about community" - has to be more specific
18	Technology	Technology (SBP3) Uses technology to accomplish and document safe healthcare delivery.	Charting, documentation, EHR transition
19	Practice-based Performance Improvement	Practice-based Performance Improvement (PBLI) Participates in performance improvement to optimize ED function, self-learning, and patient care	Well-read, EBM, good knowledge of literature, receptive to feedback, asks questions, continue to read, identifying weaknesses and improving. think about difficult cases and review learning points, identify areas you need to focus/work on, seeks learning opportunities, inquisitive
20	Professionalism	Professional values (PROF1) Demonstrates compassion, integrity, and respect for others as well as adherence to the ethical principles relevant to the practice of medicine.	Professional values, compassion (can double code w patients if mentions them), good role model, example for juniors, BUT NOT if taking active steps regarding juniors, conscentious, maturity, integrity, respect, honesty, genuine interest, tolerance, caring, any reference to behavior that is unprofessional like frustration, ?dedicted (JR votes yes)?
21	Accountability	Accountability (PROF2) Demonstrates accountability to patients, society, profession and self.	(includes outside clinical areas) wellness, don't over-work, burn-out, responsible, timely, punctual, recognizes limits, grooming, appearance, reliable, follow through, stay engaged, NOT taking on responsibility, completing responsibilites, resilience
22	Patient Centered Communication	Patient Centered Communication (ICS1) Demonstrates interpersonal and communication skills that result in the effective exchange of information in collaboration with patients and their families	bedside manner, good/interested in patient care, needs to be specific to patients (otherwise its a professional value), patient advocate, empathy, compassion for patients (double code), doing the right thing for patients
23	Team Management	Team Management (ICS2) Leads patient-centered care teams, ensuring effective communication and mutual respect among members of the team.	Works well with others, team leader, supervising juniors (NOT TEACHING), knowing which of your juniors' patients are sick, leadership, communication skills, interpersonal skills, any behavior explicitly impacting team, dealing with consultants. Actively interacts with juniors (mentoring, checking in on them), debrief, delegate, "leads the area", "good leadership of the area"
